# The association between the increased performance of laparoscopic colon surgery and a reduced risk of surgical site infection

**DOI:** 10.1007/s00595-019-1760-1

**Published:** 2019-01-25

**Authors:** Yoshinori Kagawa, Daisaku Yamada, Makoto Yamasaki, Atsushi Miyamoto, Tsunekazu Mizushima, Kazuo Yamabe, Mitsunobu Imazato, Hiroki Fukunaga, Shogo Kobayashi, Junzo Shimizu, Koji Umeshita, Toshinori Ito, Yuichiro Doki, Masaki Mori

**Affiliations:** 1 Department of Surgery, Kansai Rosai Hospital, 3-1-69 Inabaso Amagasaki, Hyogo, 660-8511 Japan; 2 Department of Gastroenterological Surgery, Graduate School of Medicine, Osaka University, 2-2 Yamadaoka, Suita, Osaka 565-0871 Japan; 3 Department of Gastroenterological Surgery, Osaka International Cancer Institute, 3-1-69 Otemae, Chuo-ku, Osaka, 541-8567 Japan; 4 Department of Surgery, Osaka Rosai Hospital, 1-1-1 Nishiku Sakai, Osaka, 591-8025 Japan; 5 Department of Surgery, National Hospital Organization Osaka National Hospital, 2-1-14 Houenzaka Chuouku, Osaka City, Osaka 540-0006 Japan; 6 Department of Surgery, Kinan Hospital, 46-70 Shinjyo-cho, Tanabe City, Wakayama Japan; 7 Department of Surgery, Osaka Police Hospital, 10-31Kitayama-cho, Tennouji-ku, Osaka City, Osaka Japan; 8 Department of Surgery, Itami City Hospital, 1-100 Koyaike Itami, Hyogo Japan

**Keywords:** Laparoscopic surgery, Surgical site infection, Colorectal surgery

## Abstract

**Purpose:**

Surgical site infection (SSI) is the most frequently occurring nosocomial infection. Remarkable surgical progress has recently been made in laparoscopic surgery. Therefore, our objective was to investigate the association between increased rates of laparoscopic colon surgery and SSI.

**Methods:**

We retrospectively investigated SSI surveillance data from July 2003 to December 2015. Two university hospitals and 25 university-affiliated hospitals participated in prospective SSI surveillance. Univariate and multivariate analyses were performed to detect significant associations.

**Results:**

We investigated 9655 colon surgeries. The year in which surgery was performed was significantly associated with the SSI rate (*p* = 0.0381). The rate of laparoscopic surgery gradually increased during the study period, and by 2012 it was routinely used for > 50% of colon surgeries. Laparoscopic surgery became a significant factor associated with reduced SSI rates compared with conventional open surgery once the performance rate of laparoscopic surgery reached > 50%.

**Conclusions:**

Increasing rates of laparoscopic colon surgery tended to be associated with a reduction in the SSI risk after surgical treatment of colonic disease. The results of this study might encourage surgeons to view laparoscopic surgical techniques as an evidence-based approach for reducing the risk of SSI.

**Electronic supplementary material:**

The online version of this article (10.1007/s00595-019-1760-1) contains supplementary material, which is available to authorized users.

## Introduction

Surgical site infection (SSI) is the most frequently occurring nosocomial infection and is associated with high morbidity, mortality, and hospital costs [1]. SSI is also an important clinical indicator of the quality of patient care and infection control [2].

The incidence of SSI has recently decreased, with several potential reasons posited. For example, awareness among medical personnel regarding how to prevent SSI has increased. We recently collected surveillance data for SSI that occurred after hepatobiliary–pancreatic surgery and evaluated predictive risk factors for SSI [3]. Advances in surgical techniques are also significantly associated with the reduced incidence of SSI. The conditions under which surgery is performed have improved, and recent evidence suggests that routine skin sterilization after surgery is no longer required [4]. In addition, there have been dramatic advances in surgical instruments and techniques, particularly in laparoscopic surgery. Numerous studies have revealed that laparoscopic surgery is associated with a lower incidence of SSI than traditional open surgery [5–7]. This may be because laparoscopic surgery does not require large incisions and thus causes less trauma. Furthermore, surgeons can use laparoscopic surgery to rapidly perform extraperitoneal procedures that carry a higher risk of infection, such as anastomosis. Among gastrointestinal surgeries, colon surgery is associated with a high rate of SSI; the overall postoperative infection rate ranges from 5.4 to 45.0% [8–11]. The incidence of SSI after colon surgery is decreasing, but how the increased use of laparoscopy has influenced the incidence of SSI remains unclear.

Therefore, our objective in the present study was to investigate the association between the incidence of SSI and the increased use of laparoscopic colon surgery from July 2003 to December 2015. Our primary hypothesis was that the increased use of laparoscopic colon surgery was associated with a decreased incidence of SSI. Our secondary hypothesis was that the increased use of laparoscopic surgery was associated only with a decreased incidence of skin SSI. Our chosen endpoints were a diagnosis of SSI [12] as defined by the National Nosocomial Infections Surveillance (NNIS) system [13] or no SSI within the study period.

## Materials and methods

### Patients

Two university hospitals and 25 university-affiliated hospitals in the Kansai area of Japan participated in prospective SSI surveillance from July 2003 to December 2015. Patients undergoing digestive surgery were enrolled in the surveillance program using the NNIS system. All patients provided their written informed consent to participate at each hospital. Before data collection began, study sessions were held to standardize the data collection method, as previously reported [3]. Nurses or surgeons (non-primary) who were members of the project team determined the presence of SSI based on definitions stated in the guidelines issued by the Centers for Disease Control and Prevention. The surveillance team collected SSI data during the patients’ hospital stay and after discharge from the hospital. Two authors (J.S. and Y.K.) oversaw the data collection and held twice-yearly feedback sessions with the participating hospitals. The infection-control staff prospectively collected surveillance data, including age, sex, surgical type, American Society of Anesthesiologists (ASA) physical status as determined by the anesthesiologist, date of surgery, operative time, surgical approach (open or laparoscopic), stoma creation (i.e., cases in which an intraoperative stoma was present, either created previously or during the present surgery; cases of emergency salvage stoma creation to address anastomotic leakage were excluded), use of intra-abdominal silk sutures (including their use for fascial closure; nonuse of silk sutures was defined as the use of other types of sutures/ligation materials throughout the entire surgery), and surgical wound classification according to the guidelines for SSI prevention [12]. Outcome variables included the development of SSI and the date on which the SSI occurred.

### Perioperative care and follow-up

All patients underwent the same protocols for perioperative care in accordance with the previously described guidelines for SSI prevention [12]. Intravenous antimicrobial prophylaxis was administered to all patients. A cephem-based antibiotic was administered prophylactically after the induction of anesthesia, and patients received an additional dose if the operation lasted > 3 h. In the operating room, hair on the surgical site was shaved after the induction of general anesthesia, and the skin was wiped with either 10% povidone iodine solution or 0.5% tincture of chlorhexidine. After surgery, the surveillance team at each hospital conducted routine follow-up, provided adequate care for the surgical site, and determined the occurrence of SSI.

### The SSI diagnosis

The primary outcome was a diagnosis of SSI as defined by the NNIS system [13]. According to the NNIS criteria, SSIs are classified as either incisional (superficial or deep) or organ/space. The criteria for a superficial incisional SSI were an infection occurring at the incision site within 30 days after the operation that involved only the skin and subcutaneous tissue and at least one of the following: incisional pain/tenderness, localized swelling/erythema/heat, purulent drainage from the incision, or microorganisms isolated by culturing fluid collected from the superficial incision. These SSIs were treated by opening the wound. The criteria for a deep incisional SSI were an infection related to the surgical procedure that occurred within 30 days after surgery and at least one of the following: purulent drainage from the deep incision; spontaneous dehiscence of the incision; or deliberate opening of the incision when the patient manifested incisional pain/tenderness, localized swelling/erythema/heat, or other symptoms of infection. The criteria for organ/space SSI were an intra-abdominal abscess without evidence of clinical anastomotic leakage (i.e., an intraperitoneal collection of pus diagnosed by ultrasonography, computed tomography, or laparotomy) and clinical anastomotic leakage. An intra-abdominal abscess near the leakage site was considered clinical anastomotic leakage.

### Statistical analyses

The univariate relationship between each independent variable and SSI was evaluated by Student’s *t* test for continuous variables and Pearson’s Chi-square test for categorical variables. Univariate and multivariate logistic regression analyses were used to identify risk factors for SSI based on the ten above-mentioned clinical factors. Variables with a *p* value of < 0.050 in the univariate analysis were included in the multivariate analysis. Relative risk was described by the estimated risk ratio with a 95% confidence interval. Two-sided *p* values were computed, and a *p* value of < 0.050 was considered statistically significant. All statistical analyses were performed with the JMP software program, version 10.0.0 (SAS Institute Inc., Cary, NC, USA).

## Results

### Patients’ background

Surveillance included a total of 41,122 abdominal surgeries: 3626 appendiceal, 4662 hepatobiliary–pancreatic, 6896 gallbladder, 9720 colonic, 855 esophageal, 9393 gastric, 4365 rectal, and 1610 small bowel surgeries. Of the 9655 colon surgeries (99.4% of all colonic surgeries; 65 cases were excluded because some data were lacking), the median (min–max) patient age was 70 (15–100) years. Colonic surgery was performed in 5330 (55.2%) men and 4325 (44.8%) women (Table 1). The median (minimum–maximum) operative time was 169 (21–1800) min. Emergency surgery was performed in 15.3% (1476 of 9655) of all surgeries. Laparoscopic surgery was performed in 30.9% (2987 of 9655) of all surgeries. The total incidence of SSI was 19.6% (1579/9655), including superficial (11.3%), deep (2.0%), and organ/space (3.1%) SSI. The number of new patients who enrolled in the surveillance system each year were as follows: 324 (2003), 676 (2004), 1003 (2005), 820 (2006), 815 (2007), 852 (2008), 545 (2009), 627 (2010), 464 (2011), 660 (2012), 993 (2013), 824 (2014), and 1052 (2015).

**Table 1:** Characteristics of patients enrolled in surgical site infection surveillance

Characteristics		*n*	(%)
Age	Years	70 (15–100)	
Sex	Male/female	5330/4325	55.2/44.8
Operating time	Min	169 (21–1800)	
Surgical wound classification	1/2/3/4	0/8526/581/546/0	0/88.3/6.0/5.7/0
ASA score	I/II/III/IV/V	1487/6640/1444/84/0	15.4/68.8/15.0/0.8/0
Emergency operation	Yes/no	1476/8179	15.3/84.7
Surgical approach	Open/laparoscopic	6668/2987	69.1/30.9
Combined resection	Yes/no	528/9127	5.5/94.5
Stoma	Yes/no	1236/8419	12.8/87.2
Silk use	Yes/no	2435/7220	25.2/74.8
SSI	Yes/no	1579/8076	16.4/83.6
Location of SSI	Superficial/deep/organ space	1093/190/296	69.2/12.0/18.7

Data are presented as the median (range) or number of patients

Classes 1, 2, 3, and 4 of surgical wound classification indicate clean, clean-contaminated, contaminated, and dirty-infected wound, respectively. Combined resection means simultaneous resection of other organs during colectomy; stoma, cases in which stoma was present during surgery either as preoperative stoma or creation of stoma (emergency creation of salvage stoma for anastomotic leakage was excluded); silk use, cases in which intra-abdominal silk suture was used

*ASA* American Society of Anesthesiologists, *SSI* surgical site infection

Univariate and multivariate analyses indicated a significant change in the SSI rate during the years in which surveillance data were collected. Univariate and multivariate analyses were also used to identify risk factors associated with SSI. Table 2 lists the risk factors that were significantly predictive of SSI. A multivariate analysis revealed that the year in which the operation was performed was a significant risk factor (*p* = 0.0318), indicating that the perioperative factors associated with SSI changed with time.

**Table 2:** Significant factors affecting occurrence of surgical site infection after colon surgery: univariate and multivariate analyses

Factors	Univariate analysis (*p* value)	Multivariate analysis (*p* value)
Operation year	0.0084	0.0318
Age	0.5847	–
Sex	0.0128	0.3542
Operating time	< 0.0001	< 0.0001
Surgical wound classification	< 0.0001	< 0.0001
ASA score	< 0.0001	0.0858
Emergency operation	< 0.0001	0.117
Surgical approach (laparoscopic or open surgery)	< 0.0001	< 0.0001
Combined resection	0.008	0.5947
Stoma creation	< 0.0001	< 0.0001
Silk use	< 0.0001	0.0002

Stoma, cases in which stoma was present during surgery either as preoperative stoma or creation of stoma (emergency creation of salvage stoma for anastomotic leakage was excluded); Classes 1, 2, 3, and 4 of surgical wound classification indicates clean, clean-contaminated, contaminated, and dirty-infected wound, respectively; combined resection, simultaneous resection of other organs during colectomy; silk use, cases in which intra-abdominal silk suture was used

*ASA* American Society of Anesthesiologists

### Increased use of laparoscopic surgery associated with decreased risk of SSI

**Fig. 1:**
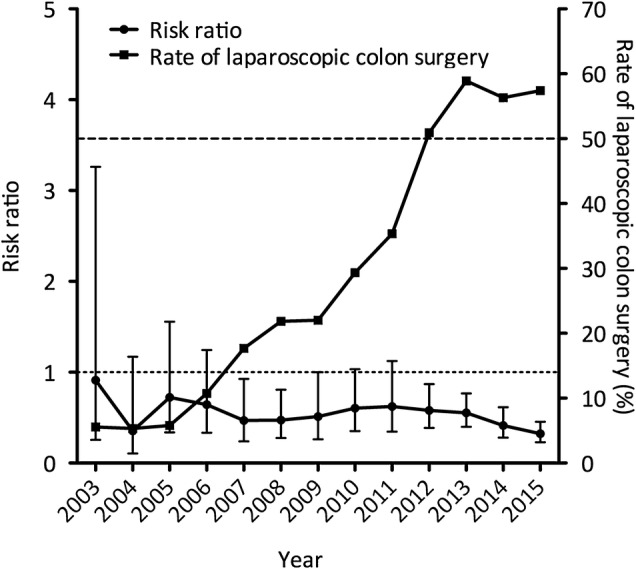
Relationship between laparoscopic surgery rate and risk ratio for the occurrence of surgical site infection after laparoscopic colon surgery. Risk ratio is denoted by circles with 95% confidence interval. Left *y*-axis, risk ratio (open surgery: laparoscopic colon surgery). Laparoscopic rate for colon surgery is denoted by squares. Right axis, percentage of laparoscopic colon surgeries (laparoscopic colon surgeries/total colon surgeries)

Univariate and multivariate analyses of the incidence of SSI were performed for each year to reveal how the risk factors changed during the study period. The identified risk factors differed from year to year (Table 3, Supplementary Table 1). Laparoscopic surgery began to emerge as a factor associated with a decreased risk of SSI from 2012 onward, although there was no such trend from 2003 to 2011. Conversely, the operating time and presence of a stoma became less significant risk factors for SSI compared with the early part of the study period (Table 3). Accordingly, we investigated the relationship between the SSI rate and the rate at which laparoscopic surgery was performed. The rate of laparoscopic surgery gradually increased, and from 2012 onward, it accounted for > 50% of colon surgeries (50.9% in 2012, 58.9% in 2013, 56.3% in 2014, and 57.4% in 2015). Figure 1 depicts the relationship between the increased use of laparoscopic surgery and SSI risk. Laparoscopic surgery became a significant factor associated with a reduced SSI risk compared with conventional open surgery once the rate at which laparoscopic surgery was performed had increased to ≥ 50%.

**Table 3:** Risk factors associated with occurrence of surgical site infection after colon surgery by year (risk ratio, *p* value)

Year	Age	Sex	Operating time	Surgical wound classification	ASA	Emergency operation	Laparoscopic surgery	Combined resection	Stoam	Silk
	< 65/≥ 65 years	M/F	< 3/≥ 3 h	1, 2/3, 4	Score I, II/III, IV, V	No/yes	No/yes	No/yes	No/yes	No/yes
2003	1.947		0.329	0.973		0.344			0.547	
	0.0314		0.0004	0.9545		0.0154			0.1792	
2004				0.303		0.934			0.348	
				< 0.0001		0.7983			0.0004	
2005			0.66	0.183	0.843	1.206			0.454	1.215
			0.0319	< 0.0001	0.4719	0.4719			0.0031	0.2709
2006			0.719	0.256	0.856					
			0.103	< 0.0001	0.5492					
2007		0.701	0.564	0.461		0.981	2.14		0.533	
		0.1111	0.0132	0.0344		0.953	0.0231		0.0473	
2008				0.374	0.585	0.515	1.438	0.631	0.519	
				0.005	0.0526	0.0602	0.1893	0.1302	0.0279	
2009		0.608		0.478		0.72	1.408		0.437	
		0.0476		0.0726		0.3692	0.3257		0.013	
2010			0.709	0.511		0.491	1.192		0.477	
			0.1661	0.0327		0.0247	0.5623		0.0198	
2011				0.273		0.766				
				0.0008		0.4881				
2012			0.733	0.516		0.83	1.431			
			0.1453	0.027		0.5186	0.1308			
2013			0.496		0.684		1.923		0.689	
			< 0.0001		0.0602		0.0002		0.0631	
2014				0.339	0.842	0.913	1.644		0.671	0.003
				0.0005	0.4839	0.7605	0.0296		0.1852	0.0032
2015				0.502		0.359	1.623		0.624	0.006
				0.0163		0.0005	0.0483		0.1039	< 0.0001

*RR* risk ratio, *ASA* American Society of Anesthesiologists, *M* male, *F* female

Stoma, cases in which stoma was present during surgery either as preoperative stoma or creation of stoma (emergency creation of salvage stoma for anastomotic leakage was excluded); Combined resection, simultaneous resection of other organs during colectomy; Silk use, cases in which intra-abdominal silk suture was used

The increased use of laparoscopic surgery was associated not only with a decrease in the risk of skin SSI but also with decreased intraperitoneal SSI rates. The data were then divided into early and late study periods (2003–2011 and 2012–2015) in accordance with laparoscopic surgery rates of < 50% and ≥ 50%. In the early study period, the increased use of laparoscopic surgery appeared to be associated with a decrease in the rate of superficial/deep SSI not but organ/space SSI. However, the increasingly common use of laparoscopic surgery in the late study period was associated with decreased organ/space SSI in addition to decreased superficial/deep SSI (Table 4). These findings suggest that routine employment of laparoscopic colon surgery may reduce the occurrence of SSI.

**Table 4:** Surgical site infection occurrence rate associated with open versus laparoscopic colon surgery

SSI		Open (%)	Laparoscopic (%)	*p* value
Total SSI	2003–2011	17.3	11.8	< 0.0001
	2012–2015	23.1	9.3	< 0.0001
Superficial/deep SSI	2003–2011	13.9	9.9	< 0.0001
	2012–2015	19.4	7.5	< 0.0001
Organ/space SSI	2003–2011	3.5	2.4	0.0964
	2012–2015	3.6	2	0.0023

*SSI* surgical site infection

The data were subsequently divided into early and late study periods (2003–2011 and 2012–2015) in accordance with laparoscopic surgery rates of < 50% and ≥ 50%. In the early study period, increased use of laparoscopic surgery appeared to be associated with a decrease in the rate of superficial/deep SSI but not but organ/space SSI. However, the increasingly common use of laparoscopic surgery in the late study period was associated with decreased organ/space SSI in addition to decreased superficial/deep SSI

## Discussion

Our analysis of patients in the Kansai area of Japan who were enrolled in the NNIS system revealed three findings: (1) Both the occurrence of SSI and the risk factors for SSI changed significantly from year to year. (2) As laparoscopic surgery became increasingly common, the incidence of SSI decreased. (3) In the late study period (2012–2015), laparoscopic surgery was significantly associated with a lower incidence of organ/space SSI, indicating a decreased rate of anastomotic leakage.

Various risk factors for SSI have been reported previously, including sex [14], body mass index [8, 15, 16], ASA score [9, 14], wound classification [9, 14, 16], operating time [8, 14, 15, 17], prophylactic antibiotic use [8], creation or closure of an ostomy [9, 15, 16], use of preoperative nonabsorbable oral antibiotics [17], smoking [8], type of suture material used for fascial closure [18], type of skin closure [18], postoperative hyperglycemia [19], and total parenteral nutrition [20]. The common risk factors in our surveillance data were the ASA score, wound classification, operating time, and presence of a stoma. However, these risk factors changed to non-risk factors and back from year to year. This suggests that the true risk factors for SSI may adjust in accordance with the changing conditions surrounding surgery. The widespread use of laparoscopic surgery is one condition that changed markedly during the study period. Laparoscopic surgery is minimally invasive and usually performed with less blood loss than open surgery. However, the operating time is longer, and the cost of surgery is higher. The advantageous features of laparoscopic surgery may contribute to a decreased risk of SSI, as suggested in a previous study [5]. Furthermore, a study that used data from a large national database yielded results similar to ours, with laparoscopic colon surgery found to be associated with a significantly lower incidence of SSI than was open colon surgery [21]. When we investigated other surgeries covered by the surveillance data, such as gastric and appendiceal surgery, the results were also similar to those of our study. Laparoscopic surgery was significantly associated with a decreased risk of SSI in any year for gastric surgery, but the rate of laparoscopic gastric surgery did not reach 50% during our study period. A decreased incidence of SSI was significantly associated with the laparoscopic approach in appendiceal surgery after the rate of laparoscopic surgery had increased to > 50% (Supplementary Figs. 1, 2). Therefore, we believe that an important reason for the reduction in the rate of SSI over the study period was the increasingly routine use of laparoscopy to perform colon surgery.

To our knowledge, this is the first study to investigate the relationship between the rate at which laparoscopic surgery is performed and the risk of SSI in colon surgery. We found that a laparoscopic surgery rate of > 50% was associated with a significant decrease in the incidence of SSI in colon surgery. The major differences between laparoscopic and open procedures are the method of access, method of exposure, and extent of operative trauma. The contributing factors to the lower SSI rates associated with laparoscopic surgery are believed to be a smaller surgical incision, decreased tissue trauma, and elimination of mechanical retraction of the abdominal wall [16]. These findings suggest that laparoscopic surgery should decrease the risk of SSI at the incision site but will not affect organ/space SSI rates because organ/space SSI is mainly caused by anastomotic leakage [22].

Some laparoscopic instruments appear to prevent anastomotic leakage; however, these instruments are also used for open surgery. We therefore expected that a comparison of open and laparoscopic surgery within the same time period would reveal a similar rate of organ/space SSI as has been reported in most previously published studies on this topic [23, 24]. Instead, the incidence of organ/space SSI was significantly different between the two methods. Some previously published findings support our results: a large multicenter sample analysis of patients undergoing colon surgery revealed that laparoscopy is associated with a lower incidence of leakage than is open surgery (1.8% vs. 2.7%, respectively) [6]. Furthermore, Murray et al. [24] showed that laparoscopic surgery is a reduced-risk factor for anastomotic leakage after adjusting for the patient’s background (odds ratio 0.69; 95% CI 0.58–0.82). The authors suggested that the laparoscopic approach may improve access for difficult mobilizations, allowing for greater bowel length and reduced tension on the anastomosis. Laparoscopic surgery may also afford better anatomical visualization in complex cases; however, why anastomotic leakage is reduced with laparoscopic surgery remains largely unclear. The laparoscopic approach lends itself to minimally invasive tissue trauma and has been shown to reduce the systemic inflammatory response compared with conventional open surgery [25]. Rettig et al. [26] stated that perioperative inflammatory conditions affect postoperative complications, including anastomotic leakage. Laparoscopic surgery performed by well-trained surgeons can reduce the postoperative C-reactive protein concentration by decreasing surgical stress; it may also decrease organ/space SSI by reducing systemic cytokine concentrations. Further investigations are necessary to determine the mechanisms by which laparoscopic surgery leads to reduced rates of organ/space SSI. Nevertheless, based on the available evidence, we highly recommend that extensive training in laparoscopic surgery be implemented to reduce SSI rates, especially in patients at increased risk of SSI, such as those of advanced age.

Our study has several limitations. This was a multicenter retrospective observational study using our surveillance data. The surgeons involved in our surveillance basically selected a surgical approach according to the current guideline at that time. Our surveillance did not include several variables that might be associated with SSI development, such as the site of resection (right, transverse, left colon), type of anastomosis (manual, mechanic), and rate of conversion to open surgery. According to a previous analysis using the 2005–2008 American College of Surgeons National Surgical Quality Improvement Program database, superficial SSI was more likely to occur in patients who underwent left-sided colectomy than in those who underwent right-sided colectomy [27]. Information on the site of resection is important because it is correlated with the incidence of infection. Unfortunately, we did not collect this information; however, we do not believe that there was a significant change in the rates of right- and left-sided colectomy from 2002 to 2015. Therefore, we do not believe that the site of resection substantially affected the incidence of superficial SSI in this study. Mechanical anastomosis was performed in most cases in our hospitals; functional end-to-end anastomosis or the double stapling technique was performed as necessary. A few cases involving manual anastomosis were included in this study because the anastomosis type is dependent on the surgeon; however, numerous randomized controlled trials and three meta-analyses have demonstrated no significant difference in organ SSI between hand-sewn and stapled anastomosis [28–30]. We therefore regarded the contamination of anastomosis types to be in an acceptable error range. Although we lack data on the exact number of conversions that occurred each year, the rate of conversion to open surgery is now less than 1% in our hospitals. Conversion cases were enrolled into the laparoscopic group in this study, but we do not believe that this substantially affected the conclusion. Further information must be collected to better evaluate the impact of laparoscopic surgery on SSI development.

The incidence rate of SSI from 2003 to 2015 was not decreased in patients who underwent laparoscopic surgery, possibly because the indications for laparoscopic colon resection have expanded to include advanced colon cancer and emergency operations. Advanced colon cancer requires radical lymph node resection and sometimes combined resection, and these procedures may be risk factors for SSI. Therefore, we considered that the expanded indications for laparoscopic surgery did not decrease the actual SSI incidence rate. We reanalyzed the odds ratio for the incidence of SSI using data that excluded combined resection and emergency operations. The resulting odds ratio was almost the same as that for the total data (Supplementary Table 3). In the latter period of the study, the indications for laparoscopic colon resection were being expanded; accordingly, open surgery was performed in more complicated cases, such as those requiring combined resection of more than two organs, organ reconstruction, or resection for local recurrence. Furthermore, the odds ratio of SSI was decreased even after the indications were expanded. Our society began to perform laparoscopic surgery for advanced colon cancer, combined resection, and emergency operations around 2010. The decrease in the odds ratio after 2011 indicates that familiarization with laparoscopic surgery reduced the risk of SSI, regardless of the indication for laparoscopic surgery.

In conclusion, we found that the widespread use of laparoscopic surgery to treat colon disease may confer protection against SSI. The results of this study should encourage surgeons to view laparoscopic surgery as an evidence-based approach to reducing the risk of SSI. Further advances in and familiarity with laparoscopic techniques will help improve patients’ clinical courses while reducing the risk of SSI. However, our study has some limitations, as the data were based on our SSI surveillance conducted according to the established guidelines at that time. Further investigations are necessary to determine the mechanisms by which laparoscopic surgery leads to reduced rates of organ/space SSI. We are now prospectively collecting SSI-related data, including the type of surgery.

## Electronic supplementary material

Below is the link to the electronic supplementary material.

Supplementary material 1 (DOCX 333 KB)

## References

[1] Poulsen KB, Bremmelgaard A, Sorensen AI, Raahave D, Petersen JV (1994). Estimated costs of postoperative wound infections. A case-control study of marginal hospital and social security costs. Epidemiol Infect.

[2] Kirkland KB, Briggs JP, Trivette SL, Wilkinson WE, Sexton DJ (1999). The impact of surgical-site infections in the 1990s: attributable mortality, excess length of hospitalization, and extra costs. Infect Control Hosp Epidemiol.

[3] Nakahira S, Shimizu J, Miyamoto A, Kobayashi S, Umeshita K, Ito T (2013). Proposal for a sub-classification of hepato-biliary-pancreatic operations for surgical site infection surveillance following assessment of results of prospective multicenter data. J Hepatobiliary Pancreat Sci.

[4] Holm C, Petersen JS, Gronboek F, Gottrup F (1998). Effects of occlusive and conventional gauze dressings on incisional healing after abdominal operations. Eur J Surg.

[5] Kiran RP, El-Gazzaz GH, Vogel JD, Remzi FH (2010). Laparoscopic approach significantly reduces surgical site infections after colorectal surgery: data from national surgical quality improvement program. J Am Coll Surg.

[6] Poon JT, Law WL, Wong IW, Ching PT, Wong LM, Fan JK (2009). Impact of laparoscopic colorectal resection on surgical site infection. Ann Surg.

[7] Aimaq R, Akopian G, Kaufman HS (2011). Surgical site infection rates in laparoscopic versus open colorectal surgery. Am Surg.

[8] Itani KM, Wilson SE, Awad SS, Jensen EH, Finn TS, Abramson MA (2006). Ertapenem versus cefotetan prophylaxis in elective colorectal surgery. N Engl J Med.

[9] Tang R, Chen HH, Wang YL, Changchien CR, Chen JS, Hsu KC (2001). Risk factors for surgical site infection after elective resection of the colon and rectum: a single-center prospective study of 2,809 consecutive patients. Ann Surg.

[10] Young H, Knepper B, Moore EE, Johnson JL, Mehler P, Price CS (2012). Surgical site infection after colon surgery: National Healthcare Safety Network risk factors and modeled rates compared with published risk factors and rates. J Am Coll Surg.

[11] Anthony T, Murray BW, Sum-Ping JT, Lenkovsky F, Vornik VD, Parker BJ (2011). Evaluating an evidence-based bundle for preventing surgical site infection: a randomized trial. Arch Surg.

[12] Mangram AJ, Horan TC, Pearson ML, Silver LC, Jarvis WR, Guideline for Prevention of Surgical Site Infection (1999). Centers for Disease Control and Prevention (CDC) Hospital Infection Control Practices Advisory Committee. Am J Infect Control.

[13] Morikane K, Honda H, Yamagishi T, Suzuki S, Aminaka M (2014). Factors associated with surgical site infection in colorectal surgery: the Japan nosocomial infections surveillance. Infect Control Hosp Epidemiol.

[14] Romy S, Eisenring MC, Bettschart V, Petignat C, Francioli P, Troillet N (2008). Laparoscope use and surgical site infections in digestive surgery. Ann Surg.

[15] Blumetti J, Luu M, Sarosi G, Hartless K, McFarlin J, Parker B (2007). Surgical site infections after colorectal surgery: do risk factors vary depending on the type of infection considered?. Surgery.

[16] Itatsu K, Sugawara G, Kaneoka Y, Kato T, Takeuchi E, Kanai M (2014). Risk factors for incisional surgical site infections in elective surgery for colorectal cancer: focus on intraoperative meticulous wound management. Surg Today.

[17] Konishi T, Watanabe T, Kishimoto J, Nagawa H (2006). Elective colon and rectal surgery differ in risk factors for wound infection: results of prospective surveillance. Ann Surg.

[18] Rasic Z, Schwarz D, Adam VN, Sever M, Lojo N, Rasic D (2011). Efficacy of antimicrobial triclosan-coated polyglactin 910 (Vicryl* Plus) suture for closure of the abdominal wall after colorectal surgery. Coll Antropol.

[19] McConnell YJ, Johnson PM, Porter GA (2009). Surgical site infections following colorectal surgery in patients with diabetes: association with postoperative hyperglycemia. J Gastrointest Surg.

[20] Alp E, Elmali F, Ersoy S, Kucuk C, Doganay M (2014). Incidence and risk factors of surgical site infection in general surgery in a developing country. Surg Today.

[21] Cima R, Dankbar E, Lovely J, Pendlimari R, Aronhalt K, Nehring S (2013). Colorectal surgery surgical site infection reduction program: a national surgical quality improvement program–driven multidisciplinary single-institution experience. J Am Coll Surg.

[22] Ojima H, Sohda M, Ando H, Sano A, Fukai Y, Ogawa A (2015). Relationship between functional end-to-end anastomosis for colon cancer and surgical site infections. Surg Today.

[23] Arezzo A, Passera R, Scozzari G, Verra M, Morino M (2013). Laparoscopy for rectal cancer reduces short-term mortality and morbidity: results of a systematic review and meta-analysis. Surg Endosc.

[24] Murray AC, Chiuzan C, Kiran RP (2016). Risk of anastomotic leak after laparoscopic versus open colectomy. Surg Endosc.

[25] Delgado S, Lacy AM, Filella X, Castells A, Garcia-Valdecasas JC, Pique JM (2001). Acute phase response in laparoscopic and open colectomy in colon cancer: randomized study. Dis Colon Rectum.

[26] Rettig TC, Verwijmeren L, Dijkstra IM, Boerma D, van de Garde EM, Noordzij PG (2016). Postoperative interleukin-6 level and early detection of complications after elective major abdominal surgery. Ann Surg.

[27] Kwaan MR, Al-Refaie WB, Parsons HM, Chow CJ, Rothenberger DA, Habermann EB (2013). Are right-sided colectomy outcomes different from left-sided colectomy outcomes?: study of patients with colon cancer in the ACS NSQIP database. JAMA Surg.

[28] MacRae HM, McLeod RS (1998). Handsewn vs. stapled anastomoses in colon and rectal surgery: a meta-analysis. Dis Colon Rectum.

[29] Lustosa SA, Matos D, Atallah AN, Castro AA (2002). Stapled versus handsewn methods for colorectal anastomosis surgery: a systematic review of randomized controlled trials. Sao Paulo Med J.

[30] Choy PY, Bissett IP, Docherty JG, Parry BR, Merrie A, Fitzgerald A. Stapled versus handsewn methods for ileocolic anastomoses. Cochrane Database Syst Rev. 2011(9):CD004320. 10.1002/14651858.CD004320.pub3 21901690

