# Supplementary material for: The association between the increased performance of laparoscopic colon surgery and a reduced risk of surgical site infection
Source: Surg Today. 2019 Jan 25;49(6):474–81. doi: 10.1007/s00595-019-1760-1 (PMC6526142; doi:10.1007/s00595-019-1760-1)
Supplement: Supplementary file 1 — Supplementary material 1 (DOCX 333 KB) [file 595_2019_1760_MOESM1_ESM.docx]

**Supplementary Figure 1**

**
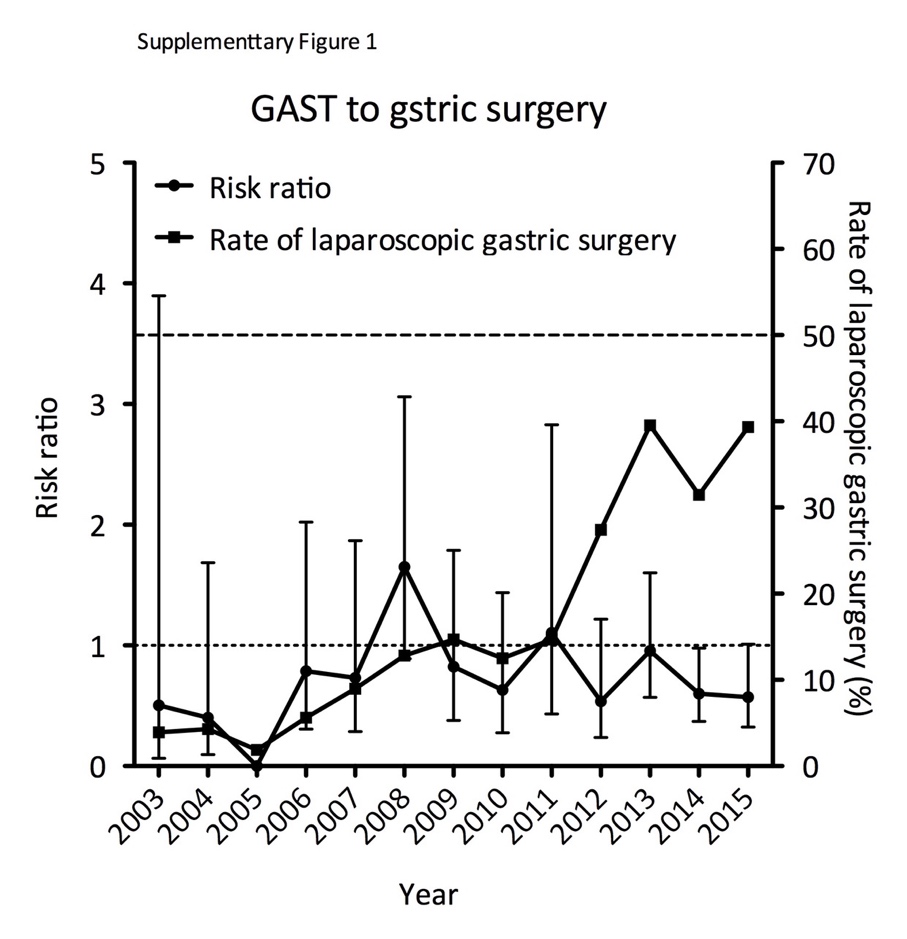
**

The relationship between the laparoscopic surgery rate and the risk ratio for the occurrence of surgical site infection after laparoscopic gastric surgery. The risk ratio is denoted by circles with 95% confidence intervals. Left y-axis, risk ratio (open surgery: laparoscopic surgery). The laparoscopic rate for gastric surgery is denoted by squares. Right axis, percentage of laparoscopic gastric surgeries (laparoscopic gastric surgeries/total gastric surgeries)

**Supplementary Figure 2**

**
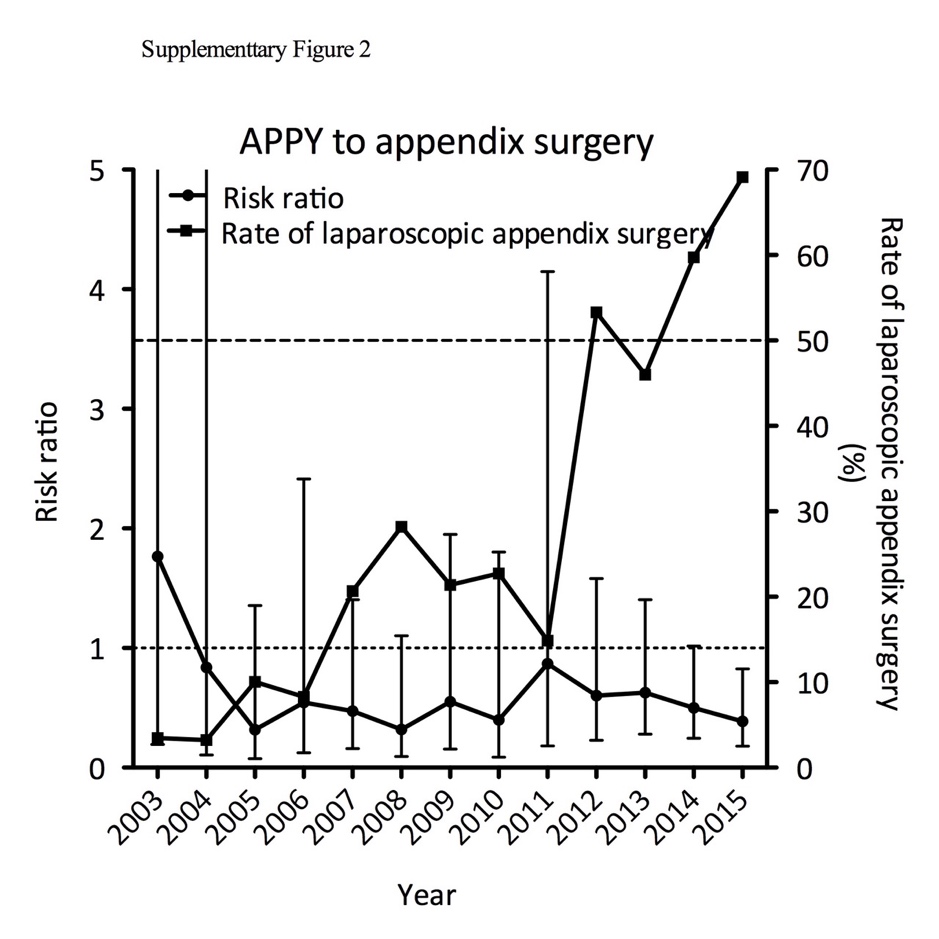
**

The relationship between the laparoscopic surgery rate and the risk ratio for the occurrence of surgical site infection after laparoscopic appendiceal surgery. The risk ratio is denoted by circles with 95% confidence intervals. Left y-axis, risk ratio (open surgery: laparoscopic surgery). The laparoscopic rate for appendiceal surgery is denoted by squares. Right axis, percentage of laparoscopic appendiceal surgeries (laparoscopic appendiceal surgeries/total appendiceal surgeries)

**Supplementary Table 1.** Univariate analysis of risk factors associated with surgical site infection development by year (*p* value)

| Year | Age | Gender | Operating time | Surgical wound classification | ASA score | Emergency surgery | Laparoscopic surgery | Combined resection | Stoma | Silk |
| --- | --- | --- | --- | --- | --- | --- | --- | --- | --- | --- |
| 2003 | 0.0064 | 0.6568 | <0.0001 | <0.0001 | 0.1596 | 0.0003 | 1.0000 | 0.1004 | 0.0128 | 0.7726 |
| 2004 | 0.0774 | 0.2404 | 0.2449 | <0.0001 | 0.0754 | 0.0163 | 0.0861 | 0.0549 | <0.0001 | 1.0000 |
| 2005 | 0.2529 | 0.5622 | <0.0001 | <0.0001 | 0.018 | <0.0001 | 0.4827 | 0.8853 | <0.0001 | 0.4582 |
| 2006 | 0.2137 | 0.3547 | 0.004 | <0.0001 | 0.0029 | 0.2604 | 0.235 | 0.3504 | 0.5019 | 0.0947 |
| 2007 | 0.5284 | 0.0439 | 0.0134 | 0.0001 | 0.3109 | 0.0153 | 0.026 | 0.0911 | 0.0011 | 0.0642 |
| 2008 | 0.3746 | 0.509 | 0.2537 | <0.0001 | <0.0001 | <0.0001 | 0.0043 | 0.0378 | <0.0001 | 0.4878 |
| 2009 | 0.1956 | 0.03 | 0.7271 | <0.0001 | 0.2981 | 0.001 | 0.0579 | 1.0000 | <0.0001 | 0.4265 |
| 2010 | 0.0884 | 0.5689 | 0.0286 | <0.0001 | 0.2124 | <0.0001 | 0.0793 | 1.0000 | <0.0001 | 0.4871 |
| 2011 | 0.7938 | 0.06 | 0.1881 | <0.0001 | 0.7582 | 0.0054 | 0.1231 | 0.7840 | 0.6474 | 0.0512 |
| 2012 | 0.5216 | 0.5399 | 0.0017 | 0.0007 | 0.1711 | 0.0134 | 0.0083 | 0.0861 | 0.1746 | 0.7258 |
| 2013 | 0.0665 | 0.1314 | <0.0001 | 0.298 | 0.0444 | 0.1374 | 0.0004 | 0.5925 | 0.0239 | 0.6383 |
| 2014 | 0.4584 | 0.2404 | 0.8528 | <0.0001 | <0.0001 | <0.0001 | <0.0001 | 0.0608 | <0.0001 | 0.0225 |
| 2015 | 0.075 | 0.8014 | 0.1311 | <0.0001 | 0.0768 | <0.0001 | <0.0001 | 0.3036 | <0.0001 | <0.0001 |

ASA, American Society of Anesthesiologists; stoma, cases in which stoma was present during surgery either as preoperative stoma or creation of stoma (emergency creation of salvage stoma for anastomotic leakage was excluded); Combined resection, simultaneous resection of other organs during colectomy; silk use, cases in which intra-abdominal silk suture was used.

**Supplementary Table 2.** Superficial, deep and organ SSIs per year

|  | sSSI (%) | |  | dSSI (%) | |  | oSSI (%) | |  | total (%) | |
| --- | --- | --- | --- | --- | --- | --- | --- | --- | --- | --- | --- |
|  | lap | open |  | lap | open |  | lap | open |  | lap | open |
| 2003 | 11.11 | 12.42 |  | 5.56 | 0.33 |  | 0.00 | 5.23 |  | 16.67 | 17.97 |
| 2004 | 5.56 | 12.97 |  | 2.78 | 3.28 |  | 0.00 | 4.22 |  | 8.33 | 20.47 |
| 2005 | 1.72 | 11.85 |  | 3.45 | 3.07 |  | 8.62 | 3.17 |  | 13.79 | 18.10 |
| 2006 | 5.68 | 9.56 |  | 1.14 | 4.10 |  | 5.68 | 4.51 |  | 12.50 | 18.17 |
| 2007 | 4.17 | 8.79 |  | 2.08 | 3.13 |  | 0.69 | 1.79 |  | 6.94 | 13.71 |
| 2008 | 5.38 | 13.96 |  | 0.54 | 0.90 |  | 3.23 | 2.70 |  | 9.14 | 17.57 |
| 2009 | 8.33 | 10.12 |  | 0.00 | 2.82 |  | 0.83 | 3.53 |  | 9.17 | 16.47 |
| 2010 | 7.61 | 9.93 |  | 0.00 | 1.35 |  | 2.72 | 4.74 |  | 10.33 | 16.03 |
| 2011 | 7.93 | 14.00 |  | 1.83 | 0.00 |  | 0.61 | 1.67 |  | 10.37 | 15.67 |
| 2012 | 10.12 | 16.36 |  | 1.79 | 1.85 |  | 2.08 | 3.70 |  | 13.99 | 21.91 |
| 2013 | 9.91 | 14.46 |  | 1.71 | 3.92 |  | 2.56 | 4.66 |  | 14.19 | 23.04 |
| 2014 | 8.62 | 19.17 |  | 0.22 | 0.28 |  | 1.29 | 1.94 |  | 10.13 | 21.39 |
| 2015 | 7.78 | 19.20 |  | 0.33 | 2.23 |  | 1.82 | 4.02 |  | 9.93 | 25.45 |

sSSI: superficial SSI, dSSI: deep SSI, oSSI: organ SSI

**Supplementary Table 3.** Odds ratios of SSI incidence of laparoscopic colectomy (dates exclude emergency and combined resection)

|  | 2003 | 2004 | 2005 | 2006 | 2008 | 2009 | 2010 | 2011 | 2012 | 2013 | 2015 |
| --- | --- | --- | --- | --- | --- | --- | --- | --- | --- | --- | --- |
| Odds　ratio | 0.440 | 0.504 | 0.804 | 0.775 | 0.629 | 0.711 | 0.907 | 0.738 | 0.658 | 0.534 | 0.393 |
| 95% CI | 0.056 | 0.150 | 0.334 | 0.383 | 0.361 | 0.353 | 0.504 | 0.383 | 0.413 | 0.373 | 0.272 |
| 95% CI | 3.453 | 1.696 | 1.938 | 1.568 | 1.098 | 1.432 | 1.630 | 1.422 | 1.049 | 0.765 | 0.568 |

CI, confidence interval
